# Study on a High Performance MEMS Infrared Thermopile Detector

**DOI:** 10.3390/mi10120877

**Published:** 2019-12-13

**Authors:** Aida Bao, Cheng Lei, Haiyang Mao, Ruirui Li, Yihao Guan

**Affiliations:** 1 School of Information technology and electronics, Beijing Institute of Technology, Beijing100081, China; baoaida@nuc.edu.cn (A.B.); liruirui513@126.com (R.L.); 2 School of Instrument and Electronics, North University of China, Taiyuan 030051, China; guanyih19@163.com; 3 Institute of Microelectronics of Chinese Academy of Sciences, Beijing 100029, China; maohaiyang@ime.ac.cn

**Keywords:** MEMS, infrared detector, thermopile, etch-stop

## Abstract

This paper presents a high-performance micro-electromechanical systems (MEMS) thermopile infrared detector. It consists of a double-end beam and a dual-layer thermocouple structure, which improves the responsivity of the detector. The etch-stop structure is integrated into the detector to prevent isotropic etching-caused damage on the device. The responsivity of the detector achieved 1151.14 V/W, and the measured response time was 14.46 ms. The detector had the potential to work as a high-precision temperature sensor and as a vacuum sensor.

## 1. Introduction

Infrared detector technology has developed rapidly due to the increasing demand for wider applications, such as thermal imaging and resource exploration in industry and civilian fields [1,2,3]. Modern infrared detectors are developed based on the infrared heat effect and the photoelectric effect. The thermal infrared detector has become a hot topic because of its advantages of uncooling at room temperature, wide spectrum response, no chopping requirements, and low cost. The thermopile infrared detector has several advantages compared to other detectors. It consists of a series of thermocouples connected in a series with each other. Therefore, compared to a thermocouple element, the thermopile device can obtain a higher output signal.

It has been reported that some thermoelectric materials can improve the performance of the Micro-electromechanical Systems (MEMS)thermal reactor infrared detector. The complementary metal oxide semiconductor (CMOS) process-compatible cantilever thermopile infrared detector was developed, which utilizes Al/n-poly Si as a thermocouple material and silicon oxide/ Silicon nitride (SiO_2_/Si_3_N_4_) as a dielectric support film material [4]. Thermo-electric materials such as Bi_2_Te_3_ and Sb_2_Te_3_ were integrated in the infrared detector to obtain high-performance [5]. Single-layer thermocouple strips (SLTS) are also adopted in the infrared detector [6]. However, the number of thermocouple strips is limited due to its size limitation and relatively low performance. Meanwhile, thermopile-based devices require a thermal isolation layer between the hot junctions and the infrared (IR) absorber area, which causes the decrease in performance.

In this paper, a MEMS thermopile infrared detector is proposed. In the detector introduced in the article, a double-end beam structure is adopted. In addition, the detector uses a double-layer thermocouple structure (DLTS), that is, the N-type thermocouple strips and the P-type thermocouple strips in the detectors are located in different planes, respectively, and the size of the detector device, based on this structure, can be further reduced while maintaining high-performance. A thermal insulation structure is applied to cold and hot junctions in the device, keeping the temperature of the device’s hot junction equal to the infrared absorption regions while keeping the temperature of the cold junction equal to the heat sink. Moreover, a novel etch stop structure is integrated into the detector to prevent over-etching during isotropic dry etching from the front, which prevents cold junctions and output electrodes from floating and causing damage in the fabrication process.

## 2. Theoretical Analysis of Infrared Detector

**Figure 1:**
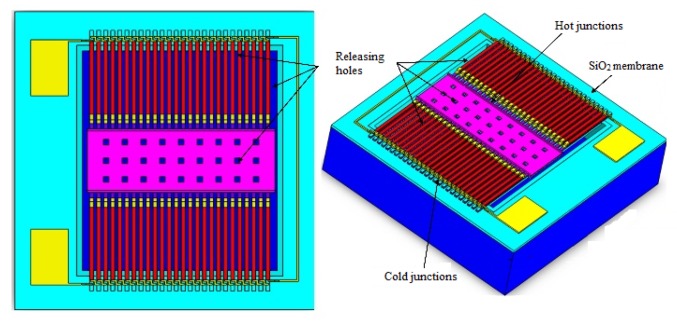
The schematic diagram of the thermopile based infrared micro-electromechanical systems (MEMS) detector.

The thermopile based infrared detector is shown in Figure 1. In the device, there is a layer of suspended dielectric film under the thermocouples, and the thermal junction of the thermopile contact with the infrared absorption zone. The cold junction of the thermocouple is located on the heat sink, which is made of silicon with good thermal conductivity. The heat sink is consistent with the ambient temperature.

When infrared radiation is applied to the thermopile device, a temperature difference (*T_diff_*) is created between the “hot junction” and the “cold junction” of the device, and according to the Seebeck effect, the thermal response voltage (Δ*U*) of the thermopile device is generated. The response output voltage of the thermopile device can be expressed as [7]:

(1) $$\Delta U=NT_{diff}(\alpha_{A}-\alpha_{B})=NT_{diff}\alpha_{AB}$$

**Figure 2:**
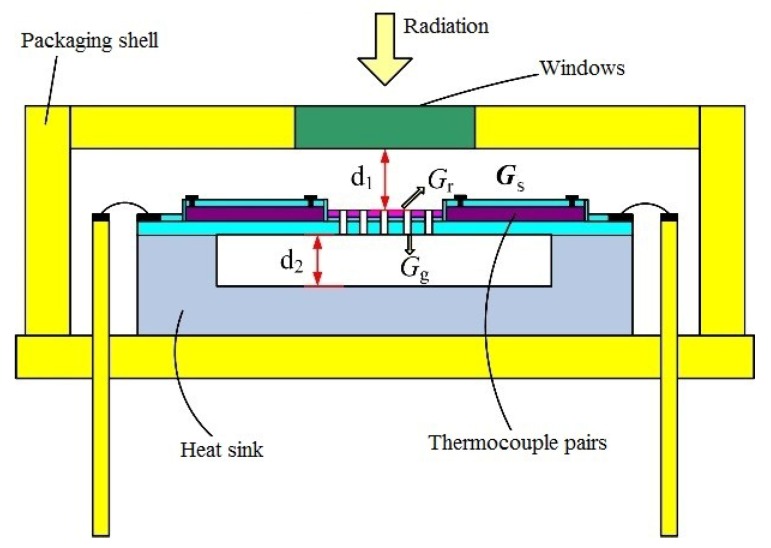
Thermal conductance distribution of the packaged thermoelectric device.

Among them, *N* is the total number of the thermocouples [7]. $\alpha_{A}$ and $\alpha_{B}$ are Seebeck coefficients of materials *A* and *B*, respectively. $\alpha_{AB}$ is the difference in the Seebeck coefficient of materials *A* and *B*. The response rate and time are important parameters in evaluating the performance of the thermopile infrared detector. The response rate of the device can be expressed as [8]:

(2) $$R_{v}=\frac{\Delta U}{P_{0}}=\frac{\Delta U}{\varphi_{0}A_{d}}$$

where *P*_0_ is the infrared radiation power, *φ*_0_ is the radiation power density, and *A_d_* is the device absorption area. According to Stefan–Boltzmann law, the power density of infrared radiation on the device surface can be expressed as [9] (assuming *T_diff_* << *T*_0_):

(3) $$\varphi_{{}_{0}}=\frac{C_{r}\cdot \sigma \cdot \varepsilon_{1}\cdot (T^{4}{}_{1}-T^{4}{}_{0})}{A_{s}\cdot \pi \cdot d_{0}{{}^{2}}_{}}$$

where *C_r_* is the root mean square conversion factor of the chopper, *σ* is the Stefan–Boltzmann constant, *ε*_1_ is the blackbody emissivity rate, *T*_1_ is the temperature of the infrared source, *T*_0_ is the ambient temperature, *A_s_* is the radiation area of the infrared source, and *d*_0_ is the distance between the infrared source and the surface of the thermopile device. In case the effect of the Joule heat and Peltier effect is neglected, the temperature difference between cold and hot junctions, *T_diff_*, can be expressed as [10]:

(4) $$T_{diff}=\frac{\eta \cdot P_{0}}{G_{th}}$$

where *η* is the infrared absorption rate of the material in the infrared absorption region and *G_th_* is the total thermal conductivity of the thermopile. As shown in Figure 2, according to energy conservation law, the infrared absorption region of the device converts the absorbed infrared radiation into heat *Q_absorb_* and transmits it through the three mechanisms: From the hot end to the cold end through the heat sink, that is, the thermal conductance of the structure, *G_s_*, the radiation thermal conductance *G_r_* in the absorption region, and the thermal conductance of the gas in the microcavity structure, *G_g_*.

(5) $$G_{th}=G_{s}+G_{g}+G_{r}$$

After substituting Equations (1) and (4) into Equation (2), the responsivity of the device can be further simplified to:

(6) $$R_{v}=\frac{N\cdot \eta \cdot \alpha_{AB}}{G_{th}}$$

Thermal conductance of the atmospheric gas and radiation are usually negligible when the device operates in a vacuum and at a low temperature. Therefore, the entire thermal conductance of the thermopile can also be written as:

(7) $$G_{th}=G_{s}$$

where *G_s_* can be expressed as:

(8) $$G_{s}=\sum_{i=1}^{4}N\frac{\lambda_{i}\cdot d_{i}\cdot w_{i}}{l_{i}}$$

Herein, $\lambda_{i}$, $w_{i}$, $d_{i}$, and $l_{i}$, respectively, are the thermal conductivity, width, thickness, and length of each thermocouple strip (*i* = 1 for the P-type thermocouple strips; *i* = 2 for the N-type thermocouple strips; *i* = 3 for the isolation layer; and *i* = 4 for the supporting membrane). Further, the responsivity of the device can be calculated by using the expression:

(9) $$R_{v}=\frac{\eta \cdot \alpha }{\sum_{i=1}^{4}\frac{\lambda_{i}\cdot d_{i}\cdot w_{i}}{l_{i}}}$$

Another important parameter is detectivity, which can be determined by:

(10) $$D^{*}=\frac{R_{v}\sqrt{A_{d}\Delta f}}{U_{n}}$$

where Δf is the measurement frequency bandwidth and $U_{n}$ is the noise voltage of the thermopile, which can be written as:

(11) $$U_{n}=\sqrt{4kR_{0}T_{0}\Delta f}$$

The noise equivalent power (NEP) can be expressed as:

(12) $$NEP=\frac{U_{n}}{R_{v}}$$

where k is the Boltzmann constant and $R_{0}$ is the electrical resistance of the thermopile strips. Then, the detectivity can be calculated as:

(13) $$D^{*}=R_{v}\sqrt{\frac{A_{d}}{4kT_{0}R_{0}}}$$

## 3. Design of the Detector

As expressed in Section 2, the performance of the thermopile detector lies in the high-difference in the Seebeck coefficient and relatively low thermal conductance of the thermocouple strips. The traditional single-layer thermocouple structure (SLTS) detector has drawbacks in regards to these two points. Herein, the thermocouple strips of the SLTS were constructed from the N-type or P-type Poly-Si and aluminum. To overcome the issues mentioned, we propose a thermopile infrared detector based on a double-layer thermocouple structure. Compared to a single-layer thermocouple structure detector, the double-layer thermocouple structure detector maintains high-performance while scaling down the size, in comparison to the single layer device. In the meantime, to avoid the over-release of the cold end of the probe and the output electrode, a novel etch stop structure is also designed in the detector.

**Figure 3:**
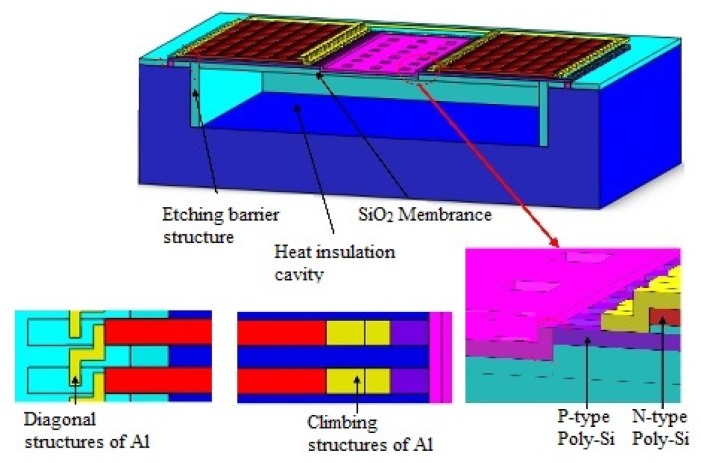
Schematic of a thermopile infrared detector based on a double-layer thermocouple structure.

In Figure 3, the detector based on the double-layer thermocouple structure adopts N-type and P-type polysilicon as the thermocouple materials, in which the N-type thermocouple and P-type thermocouple strips are located on the bottom layer and are connected in a series with each other by aluminum wires. In the hot junction of the detectors, N-type and P-type thermocouples are connected by a “climbing” aluminum structure, and the N-type and P-type thermocouple strips are interconnected by a “diagonal” shaped aluminum structure in the cold junction. This arrangement of thermocouples actually increases the detector duty cycle and reduces the lateral dimensions of the device compared to conventional four-end-beam-based thermopile devices [6]. From Table 1, it can be seen that the Seebeck coefficients of the doped polysilicon material are much larger than that of aluminum, but the thermal conductivity is lower than that of aluminum. The difference in the Seebeck coefficient of the double-layer thermocouple structure of the thermopile detector is calculated at about twice as much as the thermopile detector with aluminum and the N-type or P-type polysilicon. Meanwhile, the thermal conductance of Al is much higher than that of the poly-Si. As shown in Equation (9), the double-layer thermocouple structure (DLTS) device has more than twice the sensitivity of the SLTS device. Besides this, according to Equation (9), the structural size of the DLTS device may be further scaled down by reducing the length and maintaining the relatively higher sensitivity. The resistivity of the aluminum is also much greater than that of the polysilicon. Therefore, the resistance of a thermopile detector, based on a double-layer thermocouple arrangement, is approximately double that of the single-layer thermocouple structure detector. Meanwhile, the noise voltage of the detector based on the double-layer thermocouple structure is about the square root of two of the single-layer structure detectors, according to Equation (11). The size of the double-layer thermocouple structure can be further reduced while maintaining relatively high-performance.

**Figure 4:**
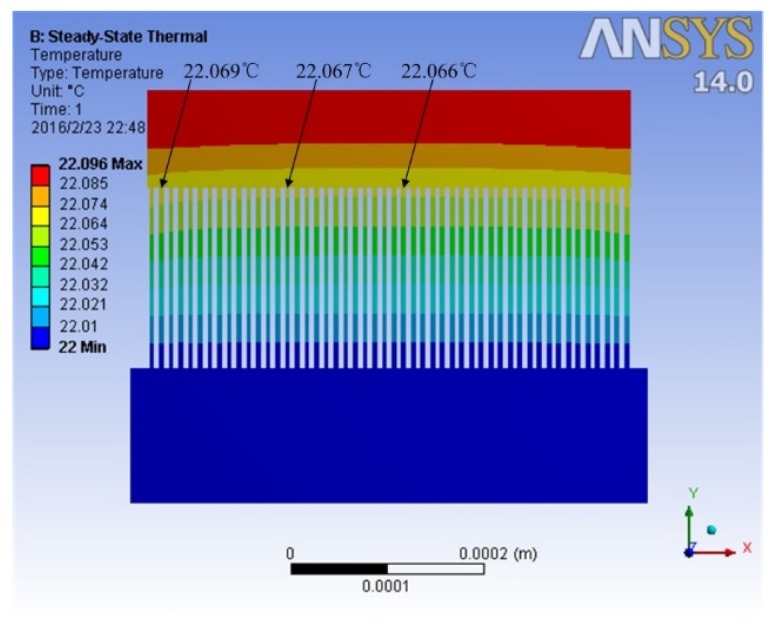
Simulation results of the temperature distribution of the detector.

The detector was modeled and simulated with Ansys workbench 14.0. The temperature of the cold junction was 22 °C, radiant power density (*φ*_0_) was 66.73 W/m^2^, and the output electrode potential of the N-type polysilicon was set as 0 V. It can be seen that the simulated temperature of the hot junction was approximately the same, and the average temperature difference between the cold junction and the hot junction was about 0.067 °C, as shown in Figure 4.

## 4. Micro-Fabrication of the Detector

The designed thermopile infrared detector with a double-layer thermocouple structure was microfabricated with the CMOS compatible process. However, isotropic dry etching of the device often leads to a suspension of the cold junction and output electrode during the release process, which causes damage to the device. To avoid this, the etch stop structure is also designed and applied in the detector.

**Figure 5:**
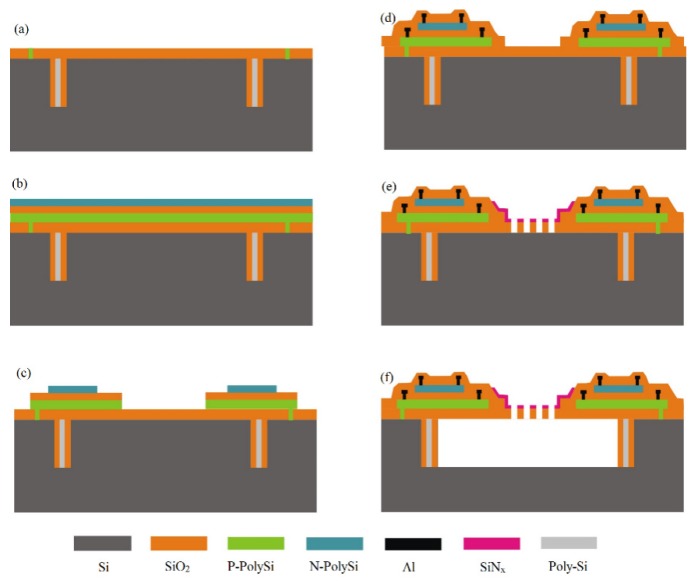
Fabrication process of the detector with a double-layer thermocouple structure and etch stop structure. (**a**) formation of etching barrier structures, (**b**) Poly-Si-SiO_2_-Poly-Si deposition and implantation, (**c**) Photo-patterning of the Poly-Si-SiO_2_-Poly-Si layers, (**d**) Passivation layer deposition afer Al patterning, (**e**) Deposition and patterning of a SiN*_x_* layer, (**f**) XeF_2_ release of Si substrate.

The micro-fabrication process of the thermopile infrared detector is shown in Figure 5. First, in order to build a release barrier structure, a deep etching process is used to form a closed-ring deep trench structure. Then, the thermal oxide and low pressure chemical vapor deposition (LPCVD) polysilicon are used to fill the deep trench, and then an 8000 Å silicon oxide dielectric support layer is grown on the wafer surface. Then, a thin film of silicon nitride with high thermal conductivity and electrically insulation is filled at the cold end. The cold end of the thermal strip is in good thermal contact with the heat sink (Figure 5a). The LPCVD process is used to deposit polysilicon, silicon oxide, and polysilicon thin films sequentially; the thickness of the polysilcon films are 5500 Å and are P-type and N-type doped, respectively. The silicon oxide film, which is located between two polysilicon films, is utilized as a thermal insulation layer with a thickness of 4000 Å (Figure 5b). The three-layer films are patterned to form a double-layer polysilicon thermocouple structure. Then, the film is re-etched, such that the top polysilicon (N-type) is shorter than the bottom polysilicon (P-type) to facilitate the connection of multiple pairs of thermocouples (Figure 5c).

After that, a layer of silicon oxide on the surface of the double-layer polysilicon thermocouples is deposited as a protective film for the structure. After the film is patterned, an ohmic contact hole in the structure is formed, and then a patterned aluminum wire layer is formed. The cold area and hot junction areas are, respectively, connected by the “diagonal” shape and the “climbing” shape aluminum line, which realizes the electrical connection between the thermocouples, as shown in Figure 3. Then, the plasma enhanced chemical vapor deposition (PECVD) process is used to grow a silicon oxide film as a passivation layer with a thickness of 4000 Å on the wafer surface. After the passivation layer is patterned, the silicon oxide layer on the surface of the hot end is removed and the hot end of the P-type thermocouple is exposed (Figure 5d). Then, a SiN*_x_* film, with a thickness of ~6000 Å, is deposited on the SiO_2_ layer and patterned into the absorber (Figure 5e). Later on, a SiO_2_ dielectric layer is further deposited by PECVD, and then, releasing windows are opened in this layer. Finally, the thermopile device is released by isotropic etching of XeF_2_ gas (Figure 5f). 

**Figure 6:**
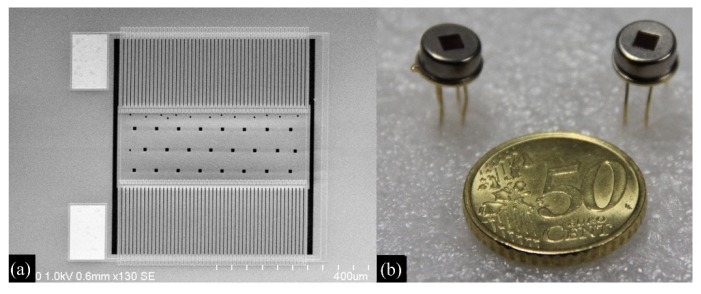
The fabricated thermopile infrared detector (**a**) and the packaged device (**b**).

The thermopile infrared detector has been successfully prepared, as shown in Figure 6a. The size of the detector is only 1.5 × 1.5 mm^2^; Figure 6b is the photo of the detector after vacuum encapsulation.

## 5. Characterization of the Detector

An infrared testing system, based on the water cooling system, was established. The water cooling system ensures that the temperature of the detector is consistent with room temperature. The testing system consists of a black body infrared radiation source (Eletrip BR 500, Optris GmbH, Berlin, Germany), a chopper (Stanford Research Systems SR540, Standford Research Systems, Sunnyvale, CA, USA), a custom water cooling system, an infrared detector, a low-pass filter circuit module, and a B1500A semiconductor characteristics analyzer (Keysight Technologies, Santa Rosa, CA, USA).

The vacuum encapsulated thermopile infrared detector is fixed in the water cooling system, and the temperature of the heat sink of the device is controlled at 22 °C. The chopper is placed between the detector and the black body to control the chopper frequency, and the low-pass filter circuit is used to suppress high-frequency noise in the test. The detector’s infrared radiation response signal is processed by a low-pass filter circuit and recorded with the semiconductor characteristics analyzer.

**Figure 7:**
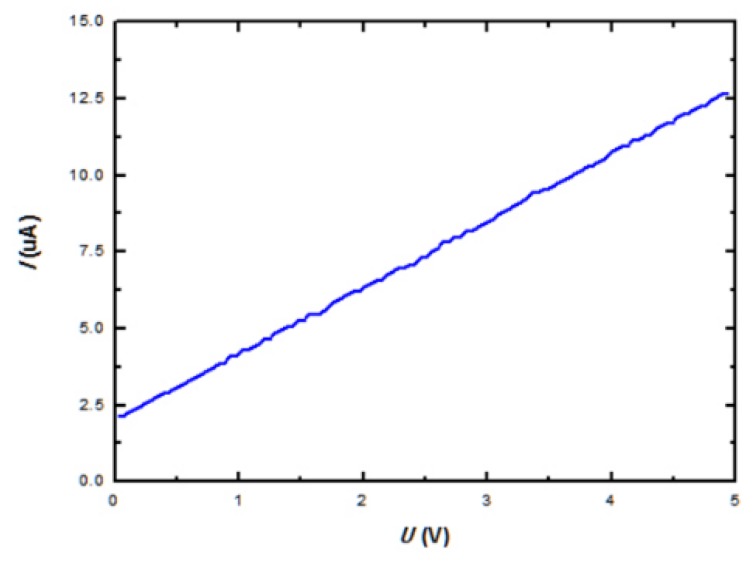
I-V characteristic curves of the thermopile infrared detector.

**Figure 8:**
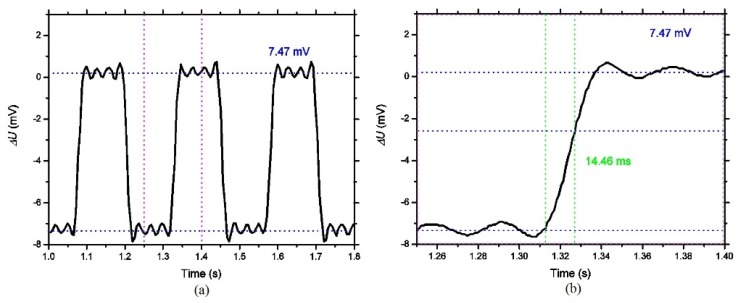
Response voltage of the detector under a black body radiation of 500 K and a chopper frequency of 4 Hz (**a**) and the enlarged rising edge (**b**).

During the test, the temperature of the blackbody is set to 500 K. At this time, the infrared radiation power density on the surface of the detector is 66.73 W/m^2^. In addition, the frequency of the chopper is set to 4 Hz. The tested result of the thermopile infrared detector is shown in Figure 7 and Figure 8. Figure 7 shows the I-V characteristic curves of the device. The tested results show that the resistance (*R*_0_) of the device is 458.5 kΩ.

Figure 8a shows the output voltage waveform of the detector in three periods. The tested results show that the response voltage of the detector is 7.47 mV under a black body radiation of 500 K and a chopper frequency of 4 Hz. Figure 8b shows the enlarged rising edge of the waveform in Figure 8, which shows that the detector response time is 14.46 ms. The responsivity *R_v_* of the device can be calculated as 1151.14 V/W. Similarly, NEPcan be calculated as 7.51 × 10^−2^ nW/Hz^1/2^, and the detectivity D* can be calculated as 4.15 × 10^8^ cm Hz^1/2^/W.

This detector can also function as a vacuum sensor and a temperature sensor with high sensitivities. The vacuum sensing capability of the device was tested with a temperature and pressure controlled cavity. The MEMS vacuum sensor is placed in the cavity, and the black body at a specific temperature is used to provide a constant heating power to the chip, so that the heat absorption area maintains a constant surface radiation power density. The vacuum pump is used to control the vacuum conditions in the chamber and the output voltage is recorded by B1500A.

**Figure 9:**
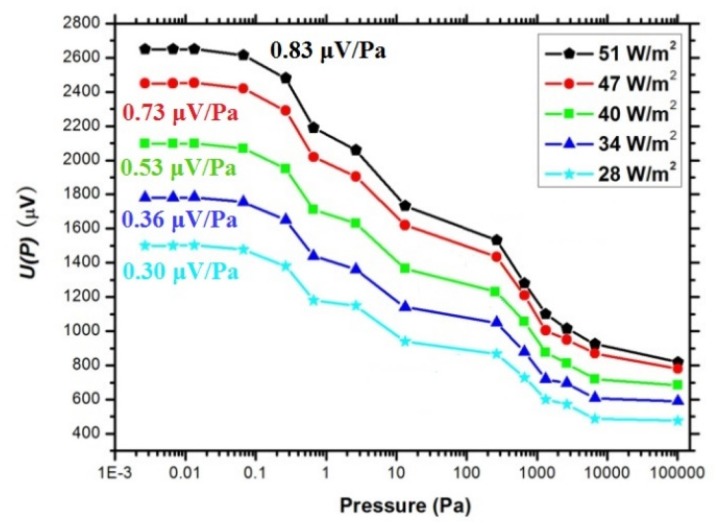
Curves for cavity pressure versus output voltage of the thermopile detector exposed to different power densities.

When the vacuum conditions in the chamber are changed, the response signals are measured. As shown in Figure 9, the output voltage of the device gradually decreases as the cavity pressure increases. We obtained the response sensitivities of the devices at the surface radiant power densities of 51, 47, 40, 34, and 28 W/m2, which are 0.83, 0.73, 0.53, 0.36, and 0.30 µV/Pa, respectively. It can be seen that, with the increase of surface radiant power density, the sensitivity of the device increases, and the upper limit of the sensitivity of the detector reaches approximately 10^5^ Pa.

**Figure 10:**
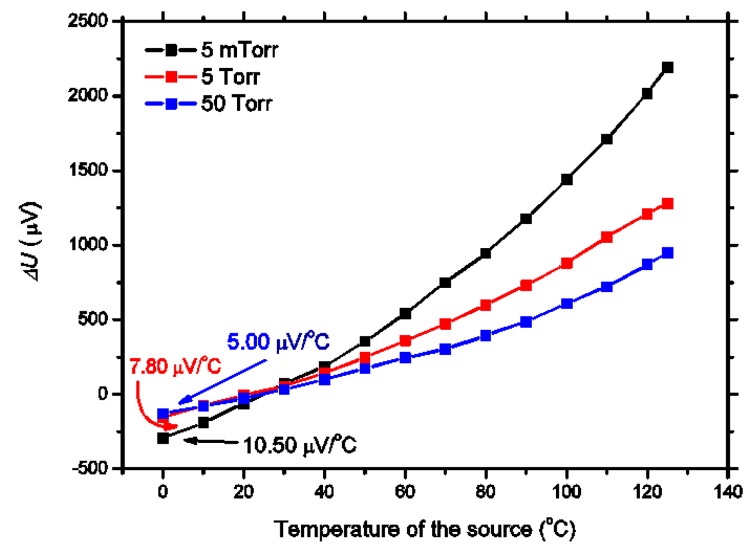
Temperature response of the thermopile infrared detector in different vacuum conditions.

The temperature responses of the thermopile infrared detectors under different vacuum conditions are tested. The vacuum pressure in the chamber is kept at a constant value of 5 mTorr, 5 Torr, and 50 Torr. When the temperature of the blackbody changes from 0 to 125 °C, the output signal of the detector is shown in Figure 10. The sensitivity of the detector with a cavity pressure of 5 mTorr, 5 Torr, and 50 Torr reaches 10.50 μV/°C, 7.80 μV/°C, and 5.00 μV /°C, respectively. The output signal of the detector increases gradually with the temperature increase. Its behavior is caused by the direct interdependence of the black body temperature and the detector surface radiation power density [6].

## 6. Conclusions

Due to the novel DLTS structure, the performance of the device has been greatly improved. A novel etch-stop structure is utilized in the detector to prevent etching damage to the structures in the releasing process. The responsivity of the sensor achieves 1151.14 V/W, NEP reaches 7.51 × 10^−2^ nW/Hz^1/2^, the detectivity D* reaches 4.15 × 10^8^ cm Hz^1/2^/W, and the response time is 14.46 ms. The response sensitivities in different temperature and vacuum conditions were measured, and can be applied in high-precision infrared detection applications. The MEMS infrared detector, with a double-layer thermocouple structure, was designed and micro-fabricated and can be integrated with CMOS circuits in the future.

## Figures and Tables

**Table 1:** Theoretical thermoelectric properties of materials [11,12].

Material Type	Al	N-Type Poly-Si (Doped @ 3.64 × 10^20^ cm^−3^)	P-Type Poly-Si (Doped @ 1.82 × 10^20^ cm^−3^)
Seebeck coefficient (μVK^−1^)	−1.66	−124.17	105.76
Thermal Conductivity (Wm^−1^K^−1^)	237	35	30
Resistivity (μΩ m)	0.03	2.7	6.55
